# Factors associated with formal and informal resource utilization in nursing home patients with and without dementia: cross-sectional analyses from the COSMOS trial

**DOI:** 10.1186/s12913-022-08675-y

**Published:** 2022-11-02

**Authors:** Maarja Vislapuu, Line Iden Berge, Renira C. Angeles, Egil Kjerstad, Janne Mannseth, Wilco P. Achterberg, Bettina S. Husebo

**Affiliations:** 1 Department of Global Public Health and Primary Care, Centre for Elderly and Nursing Home Medicine, University of Bergen, Bergen, Norway; 2 NKS Olaviken Gerontopsychiatric Hospital, Askøy, Norway; 3 NORCE Norwegian Research Centre AS, Bergen, Norway; 4 Section for Epidemiology and Medical Statistics, Department of Global Public Health and Primary Care, Faculty of Medicine, University of Bergen, Bergen, Norway; 5 Department of Public Health and Primary Care, Centre for Elderly and Nursing Home Medicine, Leiden University Medical Centre, Leiden, Netherlands; 6 Department of Nursing Home Medicine, Municipality of Bergen, Bergen, Norway

**Keywords:** Dementia, Nursing home, Formal care, Informal care

## Abstract

**Objectives:**

To investigate the association between clinical, demographic, and organizational factors and formal (health professionals) and informal (relatives) resource utilization in nursing home patients with and without dementia.

**Methods:**

Baseline data from the multicomponent cluster randomized control COSMOS trial including 33 Norwegian nursing homes and 723 residents with and without dementia. Nursing home staff (*n* = 117) participated as proxy raters to approximate formal and informal resource use in daily care.

**Measurements:**

The primary outcome was the Resource Utilization in Dementia - Formal Care scale to assess formal and informal care time in hours/month regarding basic activities of daily living (ADL), instrumental ADL, and supervision. Secondary outcomes were hours/week spent on formal and informal leisure activities. Behavioral and psychological symptoms in dementia (BPSD) were assessed by the Neuropsychiatric Inventory-Nursing Home version, physical function by the Physical Self-Maintenance Scale, and psychotropic drug use by the Anatomical Therapeutic Chemical classification system. Organizational factors were ward size and staff ratio.

**Results:**

Generalized linear mixed-effect models and two-part modelling revealed an association between increased formal care time and poorer physical function, higher agitation and psychotropic drug use and lower cognitive function (all *p* < .05). Enhanced formal leisure time was related to better ADL function (*p* < .05) and smaller wards (*p* < .05). The family related leisure time was associated with agitation, decline in ADL function, smaller wards, and better staffing ratio (all *p* < .05). Married patients received more informal direct care (*p* < .05) and leisure time (*p* < .05) compared to unmarried/widowed.

**Conclusion:**

For nursing home staff, higher agitation and psychotropic drug use, and lower cognitive function, is associated with more direct care time, whereas leisure time activities are less prioritized in people with lower physical function. Informal caregivers’ engagement is encouraged by smaller nursing homes and better staff ratio. Therefore, we recommend stakeholders and healthcare professionals to consider these clinical and organizational factors to optimize treatment and leisure time activities in nursing home patients with various needs.

**Trial registration:**

ClinicalTrials.gov; NCT02238652.

**Supplementary Information:**

The online version contains supplementary material available at 10.1186/s12913-022-08675-y.

## Background

Dementia is a costly disease with rapidly increasing indirect and intangible expenditures [1]. Resource utilization in dementia care is mainly driven by informal caregiving (relatives) and formal caregiving (health professionals) [1]. Most people with dementia are cared for at home by informal caregivers living with them or living nearby [2]. In the later stages of the disease, institutionalization in nursing homes (NHs) is common practice in many Western countries [3]. Behavioral and psychological symptoms in dementia (BPSD), care dependency, and multimorbidity are the main reasons for NH admission [4]. This transition has considerable impact on care hours provided by different services. For instance, informal care by relatives has shown to diminish from 160 hours per month at home to approximately 7–9 hours/month in the NH [5–7], while formal care provided by homecare services increases from 18 hours/month to 76 hours provided by the NH staff [6, 8].

Most NH patients in Norway have cognitive impairment and more than 80% meet the diagnostic criteria for dementia [9]. Even when under formal care, the support and attention by family members is still crucial to safeguard the person’s autonomy, quality of life (QoL), and care [10]. A quasi-experimental study from the Netherlands (2005) demonstrates the supportive value of relatives in activities of daily living (ADL) such as personal hygiene and food intake [11]. Further, family members oversee and manage daily care, provide socioemotional support, and act as complements in leisure activities [12]. Meanwhile, a comprehensive cross-sectional study by Roberts et al. (2020) suggests that male spouses visit more often than female spouses, however female (spouses and children) deliver more direct care. The visitation frequency increased with the patients` cognitive function, whereas the care provision was associated with lower cognitive functioning [13]. Furthermore, relatives contribute to psychological stability, shared decision making, better care quality [10], and timely detection of changes in health [14].

While informal care time in NHs is considered important for care quality and patient outcomes, studies focusing on clinical and organizational factors related to informal caregiving are limited. The association between total care time in NHs and cognitive function was investigated by Nordberg et al. (2007), showing variations in time use due to dementia and differences in ADL dependency [15]. Another study by Buylova et al. (2020) demonstrated that agitation caused by advanced dementia is the main determinant for formal and informal care resources utilization [16]. However, analyses in these studies forgo other factors such as additional BPSD to agitation, ward size, and staffing [17].

The COSMOS trial aimed to develop, implement, and evaluate the effect of a multicomponent intervention on QoL in NH patients with and without dementia [18, 19]. COSMOS is the acronym for COmmunication, Systematic pain assessment and treatment, Medication review, Organization of activities, and Safety. In this study, we analyze the baseline data from the trial and explore the cross-sectional association between clinical, demographic, and organizational factors, to identify formal and informal resource utilization in connection with time spent on direct care and leisure activities. We hypothesize that:

1) increased formal direct care time in NHs is associated with higher BPSD score and lower physical and cognitive functioning;

2) formal direct care time by NH staff is positively associated with informal direct care time by relatives;

3) informal leisure time is negatively associated with ward size and staffing ratio.

## Methods

### Study population and data collection

This study used baseline data from the 9-month, cluster-randomized control COSMOS trial (May 2014 – December 2015), including 33 Norwegian NHs, aimed to improve the patients’ QoL. Clinicaltrials.gov Identifier: NCT02238652, registered 12/09/2014. The intervention lasted for 4 months with assessments and data collection performed at baseline, at month four, and at follow-up after 9 months. NH staff (*n* = 117) who previously participated in a two-day education seminar completed the data collection [18, 19]. The inclusion criteria were ≥ 65 years old, life expectancy ≥6 months (to prevent early drop-out of the patients) and residing in long-term care or specialized dementia units.

### Primary and secondary outcome measures

The primary outcome was the Resource Utilization in Dementia – Formal Care tool (RUD-FOCA) that assesses formal and informal direct care time in hours per month spent on basic activities of daily living (ADL) functioning (e.g., toileting, personal hygiene, and meal situations) and instrumental ADL (IADL) (e.g., taking medicine, out-patient visits) in NHs. The RUD-FOCA also consists of monthly hours of supervision such as surveilling dangerous events and providing guidance [20]. The RUD-FOCA is widely used, allowing for comparisons across countries and continents [21]. The tool has shown good retest-reliability and construct validity, and been translated into different languages, including Norwegian, and is being used in clinical trials [17, 20, 21]. The secondary outcome was formal and informal leisure time assessed in hours/week to cover physical (e.g., walking outside, group exercise), mental (e.g., reminiscence, bingo), and social (e.g., performance, music) activities.

### Covariates

The severity of dementia was measured by the Mini-Mental State Examination (MMSE). The MMSE is a 30-point scale with lower scores indicating lower cognitive function (≥24 = no impairment, 18–23 = mild dementia, 12–17 = moderate dementia, 0–11 = severe dementia) [18, 22]. The severity and frequency of BPSD was assessed by the Neuropsychiatric Inventory-Nursing Home Version (NPI-NH), which has shown good internal consistency (α > 0.8) and high inter-rater reliability (0.85–1.0) in the Norwegian NH population [23]. NPI-NH includes 12 domains of BPSD over the past 4 weeks and can be clustered into mood (depression, anxiety, apathy, sleep, and appetite), agitation (agitation, disinhibitions, irritability, and aberrant motor behavior), and psychosis (hallucinations and delusions) clusters [18]. Total score range from 0 to 144; a higher score indicates more symptoms. The 6-item Physical Self-Maintenance Scale (PSMS) (range 6–30) was used to measure the level of physical functioning (showering, toileting, eating, and mobility), with a higher score indicating lower functional capacity. The PSMS scale has shown high inter-rater reliability (0.91) and good validity [24]. The number of regular psychotropic drugs was included according to the Anatomical Therapeutic Chemical Index (antipsychotics, anxiolytics, hypnotics/sedatives, antidepressants, and anti-dementia medication) [25]. Organizational factors were ward size and staff ratio. The staff ratio (including nurses, auxiliary nurses, and care assistants) on the ward was calculated as follows [8]:

$$\frac{\mathrm{staff}\;\mathrm{ratio}\;=\;\left(\mathrm{no}.\;\mathrm{staff}\;\mathrm{at}\;\mathrm{daytime}\;+\;\mathrm{evening}\;\mathrm{on}\;\mathrm{weekdays}\right)\ast5\;+\;\left(\mathrm{no}.\;\mathrm{staff}\;\mathrm{at}\;\mathrm{daytime}\;+\;\mathrm{evening}\;\mathrm{in}\;\mathrm{weekends}\right)\ast2}{\mathrm{number}\;\mathrm{of}\;\mathrm{residents}\;\mathrm{on}\;\mathrm{the}\;\mathrm{ward}}$$

### Statistics

Descriptive statistics are shown as means, standard deviations (SD), and percentages. The outcome of the generalized linear mixed-effect models (GLMM) was hours/month of formal direct care (Table 2, Model 1) and hours/week in formal leisure time (Table 2, Model 2). A high degree of non-normality was observed in both outcome variables, suggesting that a generalized linear model (GLM) was appropriate [26]. In the presented model, a random effect was included in the GLMM based on the possible cluster effect of the NHs. Due to the logarithmic link function used in the GLM, the estimated effects are expressed as the relative change in resource use due to an increase in the covariates. A small number of non-bedridden patients were registered with zero hours of formal care per month. We considered that all NH patients receive some amount of formal direct care and social interaction, thus 0.1 hours of formal care/month were added to each of the zero-hour cases.

The outcomes of the two-part models were informal direct care (Table 3, Model 1) and informal leisure time (Table 3, Model 2). We applied a two-part model due to the structure of the data with zero observations as true zeros and the actual time received being continuous and positively distributed non-zero values [27]. The binary part of the model was estimated using logistic regression with informal direct care/leisure time (yes or no) as the binary variable, while the non-binary part of the model was estimated using a gamma GLM. Hours of informal direct care time > 0 (Table 3, Model 1) and informal leisure time > 0 (Table 3, Model 2) were the outcome variables. The possible cluster effect of the NHs was included to allow for intragroup correlation on the NH level. The results are presented as marginal effects for the combined logistic and gamma GLM two-part model. The β-coefficients and odds ratio (ORs) of the covariates for the logit and GLM models are presented with 95% confidence intervals (CIs). The Akaike Information Criterion [28] and Hosmer–Lemeshow goodness of fit analysis were used for variable and model selection. All analyses were performed using Stata IC 16. Results were considered statistically significant for *p* < .05, and missing values were addressed using listwise deletion.

## Results

**Fig. 1:**
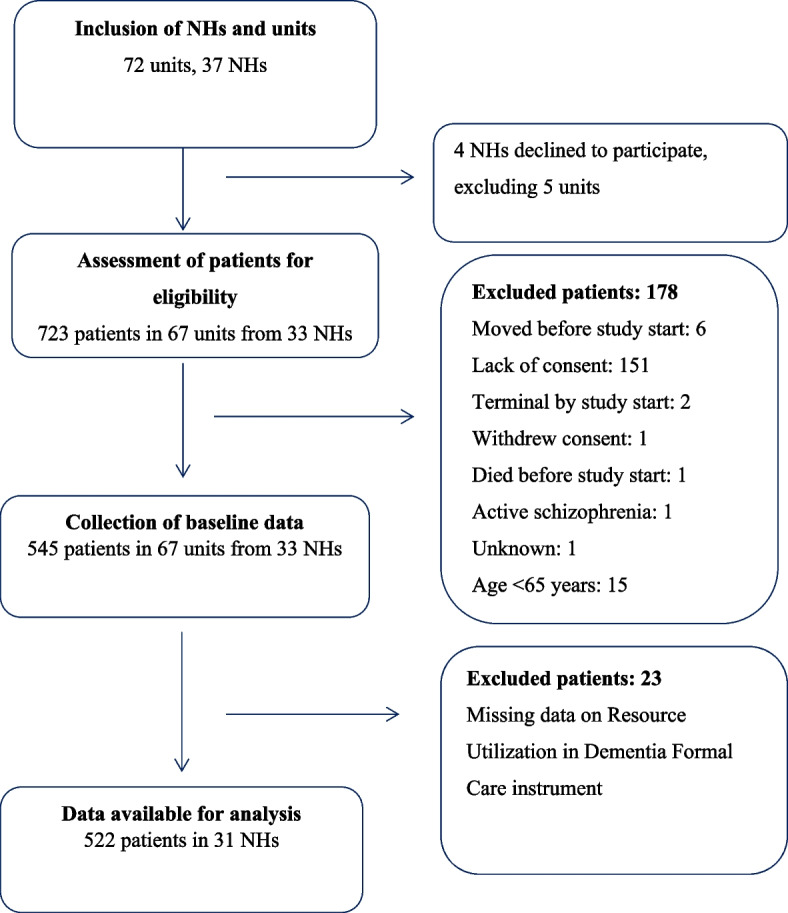
Flow Chart of Study Participants in COSMOS trial

In total, 723 NH patients were screened for participation in the COSMOS trial. One-hundred and seventy-eight patients did not complete the data collection, and 23 patients were not included due to missing data on RUD-FOCA (Fig. 1). The final study sample included 522 participants with a mean age of 86.6 years (SD 7.5), and 74% were female and 23% were married. The mean MMSE score was 10.8 (SD 7.8), and 52% (*n* = 251) had severe dementia (score 0–11); 26% (*n* = 123) had moderate dementia (score 12–17); 16% (*n* = 78) had mild dementia (score 18–23), and 6% (*n* = 28) had no dementia (score ≥ 24). The mean PSMS score was 17.3 (SD 5.4) (Table 1). Polypharmacy was common, and 79% used five or more drugs permanently, 73% were treated with one or more psychotropic drugs, and the most frequently prescribed were antidepressants (40%). Patients received a mean of 54.8 hours (SD 51.1) of formal direct care and 7.1 hours (SD 14.3) of informal direct care per month. Two-hundred and nineteen patients (42%) had complete data on activity measures. On average, total activity hours were 9.1 hours/week (SD 13.0), and informal caregivers contributed with 2.3 (SD 5.1) hours/week. The mean number of beds per ward was 18, and one staff member was responsible for approximately four patients. The table comparing demographic and clinical differences between patients with missing and complete data on leisure time is presented in Appendix 1.

**Table 1:** Characteristics of the Study Participants (*N* = 522)

	All patients (***N*** = 522)	Missing, n^a^
Age in years, mean (SD)	86.6 (7.5)	2
Female gender, n (%)	388 (74.3)	0
Married, n (%)	118 (22.6)	41
Psychotropic medication^b^ regular, 1 or more drug, n (%)	382 (73.2)	0
Polypharmacy^c^, n (%)	411 (78.7)	0
MMSE^d^, mean (SD)	10.8 (7.8)	42
PSMS^e^, mean (SD)	17.3 (5.4)	0
**NPI clusters**^f^**, mean (SD)**		
Mood cluster (0–60)	7.5 (9.7)	2
Psychosis cluster (0–24)	2.5 (4.9)	1
Agitation cluster (0–48)	7.2 (9.8)	0
**Direct care time (hours/month)**^g^		
Mean hours of formal care, mean (SD)	54.8 (51.1)	0
Receiving informal general care > 0 hours/month, yes, n (%), mean (SD)	215 (41.2)	0
Mean hours of informal care, *n* = 215, mean (SD)	7.1 (14.3)	0
**Leisure time (hours/week)**^h^		
With family or friends, (SD)	2.3 (5.1)	299
With staff, (SD)	7.0 (11.8)	303
Total time spent on leisure time, (SD)	9.1 (13.0)	296
**Organizational variables**^i^		
Number of beds, mean (SD)	17.9 (7.2)	2
Staff ratio, mean (SD)	3.8 (1.1)	2

Table legend: *N* total sample, *n* number of patients, *SD* standard deviation

^a^Missing data are reported for the total sample

^b^According to The Anatomical Therapeutic Chemical Index (antipsychotics, anxiolytics, hypnotics/sedatives, antidepressants, and anti-dementia agents)

^c^5 or more drugs regularly

^d^MMSE: Mini-Mental State Examination [range 0–30], higher scores indicate better cognition

^e^PSMS – Physical Self-Maintenance Scale [range 0–30], higher score indicates lower physical function

^f^NPI-NH: Neuropsychiatric Inventory – Nursing Home Version. Mood cluster (depression, anxiety, apathy, sleep, and appetite); agitation cluster (agitation, disinhibitions, irritability, and aberrant motor behavior), and psychosis cluster (hallucinations and delusions)

^g^Care time provided in ADL (e.g., toileting, personal hygiene, and meal situations), IADL (e.g., taking medicine, out-patient visits), and supervision (e.g., preventing dangerous events, guiding)

^h^Time registered in social activities, physical activities, and mental activities

^i^Two nursing home had missing data

### Formal and informal direct care and associations with covariates

Table 2 shows the estimates from the GLMM model analysis. Model 1 estimates showed that higher formal direct care time was associated with reduced physical function (Exp(β) = 1.07, *p* < .05; relative increase 7.2%) and with higher BPSD such as aberrant motor activity, agitation, and irritability (Exp(β) = 1.02, *p* < .05; relative increase 1.9%). The greatest increase (relative increase 31%) in formal direct care was seen in combination with psychotropic drug use (Exp(β) = 1.33, *p* < .05). Less formal direct care time was associated with higher cognitive function (Exp(β) = .98, *p* < .05; relative increase 1.7%). Including NHs as a random effect improved the AIC for the model, thus indicating differences between institutions. In the combined two-part regression model (Table 3, Model 1), married patients received more informal direct care time (95%CI .54; 8.11, *p* < .05) than patients who were not married or who were widowed.

**Table 2:** Factors Associated with Formal Direct Care Time (Model 1) and Formal Leisure Time (Model 2)

Variable	Model 1 Formal direct care time^a^ (*N* = 388)		Model 2 Formal leisure time^b^ (*N* = 164)	
	Exp (β)	*P*	Exp (β)	*P*
Informal leisure time^b^	Not included in the model		1.00	.98
Informal direct care time^a^	1.00	.21	Not included in the model	
Gender, female, (ref = male)	.95	.56	1.25	.30
Age	1.00	.24	.98	.15
Married, (ref = not married/widowed)	1.07	.48	1.43	.13
MMSE^c^	.98	**<.05**	.98	.36
PSMS^d^	1.07	**<.05**	.93	**<.05**
**NPI-NH**^e^				
Psychosis cluster				
Mood cluster	.99	.69		
Agitation cluster	1.02	**<.05**		
**Medication:**				
Polypharmacy^f^, yes				
Psychotropic medication, regular, (ref = 0 drugs)	1.33	**<.05**		
**Organizational variables**				
Number of beds	.99	.83	.95	**<.05**
Staff ratio	1.09	.11	1.33	.14

Table legend: Exp(β) is estimated with generalized linear mixed-effect models (GLMM) and interpreted as the relative change in resource use due to a one unit increase in the explanatory variable

^a^Direct care time provided in ADL (e.g., toileting, personal hygiene, and meal situations), IADL (e.g., taking medicine, out-patient visits), and supervision (e.g., preventing dangerous events, guiding)

^b^Time registered in social activities, physical activities, and mental activities

^c^MMSE: Mini-Mental States Examination [range 0–30], a lower score indicates greater cognitive impairment, and a score ≤ 20 is characteristic for dementia

^d^PSMS – Physical Self-Maintenance Scale [range 0–30], a higher score indicates lower functional capacity

^e^NPI-NH: Neuropsychiatric Inventory – Nursing Home Version. Mood cluster (depression, anxiety, apathy, sleep, and appetite); agitation cluster (agitation, disinhibitions, irritability, and aberrant motor behavior), and psychosis cluster (hallucinations and delusions). Higher scores indicate more neuropsychiatric symptoms

^f^Five or more drugs regularly

**Table 3:** Factors Associated with Informal Direct Care Time (Model 1) and Informal Leisure Time (Model 2)

	Model 1 Informal direct care time^a^			Model 2 Informal leisure time^b^		
	Logit OR (95% CI)	GLM b (95% CI)	Combined marginal effect (95% CI)	Logit OR (95%CI)	GLM b (95%CI)	Combined marginal effect (95% CI)
Age	.98 (.96; 1.00)	.00 (−.04; .04)	−.03 (−.16; .10)	1.05 (.99; 1.09)	.01 (−.02; .04)	.04 (−.03; .12)
Gender, (ref = male)	.82 (.47; 1.44)	.20 (−.39; .80)	.28 (− 1.78; 2.34)	.60 (.14; 2.56)	.15 (−.43; .73)	.11 (− 1.3; 1.53)
Married, (ref = not married/widowed)	1.29 (.73; 2.29)	.95 (.33; 1.57)*	4.33 (.54; 8.11)*	2.10 (.49; 9.00)	.46 (.10; .81)*	1.6 (.14; 3.01)*
**Clinical variables**						
MMSE^c^	1.00 (.95; 1.06)	−.06 (−.11; −.02)*	−.20 (−.43; .04)	.95 (.89; 1.01)	.03 (−.01; .07)	.05 (−.05; .15)
PSMS^d^	.99 (.93; 1.05)	−.07 (−.13; −.01)*	−.24 (−.55; .08)	.95 (.87; 1.03)	.05 (.02; .08)*	.09 (.01; .18)*
NPI-NH^e^						
Mood	.99 (.97; 1.02)	−.02 (−.04; .00)	−.07 (−.17; .02)	.99 (.93; 1.05)	−.01 (−.05; .02)	−.03 (−.12; .06)
Agitation	1.02 (.99; 1.06)	−.00 (−.03; .02)	.04 (−.07; .15)	1.08 (.99; 1.18)	.03 (.00; .07)*	.12 (.04; .20)*
Psychosis				.93 (.79; 1.09)	−.02 (−.09; .04)	−.09 (−.26; .08)
Polypharmacy^f^	.73 (.39; 1.35)	1.01 (.17; 1.84)*	2.09 (−.14; 4.32)			
Psychotropic medication use, regular, (ref = no)				1.36 (.65; 2.87)	.31 (−.09; .70)	.80 (−.06; 1.66)
**Organizational variables**						
No. of beds in the ward	1.00 (.96; 1.04)	−.00 (−.04; .03)	−.01 (−.14; .12)	1.02 (.92; 1.12)	−.05 (−.06; −.03)*	−.09 (−.15; −.04)*
Staff ratio	.72 (.47; 1.10)	−.02 (−.44; .41)	−.69 (− 2.26; .87)	.94 (.65; 1.36)	−.34 (−.58; −.09)*	−0.81 (− 1.40; −.24)*
Goodness of fit *p*-value	.31			.19		
N	388	141		64	159	

Table legend: *p < .05; *CI* confidence interval, *OR* odds ratio, *GLM* generalized linear regression

^a^Direct care time provided in ADL (e.g., toileting, personal hygiene, and meal situations), IADL (e.g., taking medicine, out-patient visits), and supervision (e.g., preventing dangerous events, guiding)

^b^Time registered in social activities, physical activities, and mental activities

^c^MMSE: Mini-Mental State Examination; a lower score indicates greater cognitive impairment, and a score ≤ 20 is characteristic for dementia

^d^PSMS – Physical Self-Maintenance Scale [range 0–30]; a higher score indicates lower functional capacity

^e^Neuropsychiatric Inventory – Nursing Home Version. Mood cluster (depression, anxiety, apathy, sleep, and appetite), agitation cluster (agitation, disinhibitions, irritability, and aberrant motor behavior), and psychosis cluster (hallucinations and delusions). Higher scores indicate more neuropsychiatric symptoms

^f^Five or more drugs regularly

### Formal and informal leisure time and associations with covariates

The informal leisure time contribution by relatives (Table 3, Model 2) was greater for patients who were married (Coef. 1.6, 95%CI .14; 3.01, *p* < .05), who had lower physical function (Coef. .09, 95%CI .01; .18, *p* < .05), and who scored higher on agitation cluster symptoms (Coef. .12, 95% CI .04; .20, *p* < .05). Formal leisure time was less for patients with reduced level of functioning (Exp(β) = .93, *p* < .05). Concerning the organizational variables, longer formal (Exp(β) = .95, *p* < .05) and informal leisure time (Coef. –.09, 95%CI −.15; −.04, *p* < .05) was associated with smaller NH wards. An increasing staff ratio was associated with decreased informal leisure time (Coef. –.81, 95%CI –1.40; −.24, *p* < .05).

## Discussion

This study aimed at elucidating the cross-sectional relationship between clinical, demographic, and organizational factors and formal and informal resource utilization in NH patients. Supporting our first hypothesis, we found that higher levels of agitation, psychotropic drug use, and lower cognitive and physical functioning were independently associated with increased direct care time by NH staff. Patients residing in larger wards and those with poor physical health received less formal leisure time. Married patients received more total informal care, whereas increased informal leisure time was associated with higher agitation and poorer physical health. Concerning our second hypothesis, we found a positive non-significant correlation between formal and informal care. However, one previous Norwegian study found that NH patients who received more than three hours of informal care also received more care form the NH staff [29]. The third hypothesis was partly confirmed, as family engagement in activities decreased in larger NH wards. Our results are of key importance for stakeholders and clinicians because creating smaller wards with user-friendly environments, in addition to better staffing can support family involvement and user-involvement.

This article extends the traditional method of estimating formal and informal NH care based on ADL and supervision task calculations [7, 15, 17]. We found that 7 hours of informal direct care per month in Norwegian NHs is relatively low compared to other European countries. For instance, a study from the UK showed that family members are engaged for 15 hours [16], and in the Netherlands they spend approximately 36 hours (9 hours/week) on informal direct care/month [7]. However, the country variations between informal direct care estimations may also be related to different data collection methods.

Although high levels of care time are a crucial objective for high-quality care, Norwegian studies demonstrate a considerable lack of physical and leisure activities provided by NH staff [30, 31]. It has also been suggested that the Coordination Reform (2012) aggravated the situation, resulting in NHs being more treatment-focused [30]. Because all public resources are limited and challenged by demographic developments, informal care engagement is a crucial substitute for formal care [32, 33]. Our study showed that larger wards and higher patient to staff ratios are associated with lower informal engagement in leisure activities. As for spousal dyads, decreased informal care in larger wards could be explained by Førsund et al. (2016), who demonstrated that spousal caregivers’ privacy in Norwegian NHs is nearly non-existent [34]. Lack of safe spaces and locked doors are challenges for leisure activities with a multidisciplinary approach. The environmental design of elderly institutions plays an important role in patients’ level of physical activity. NHs are often hospital-like and are filled with medical equipment with limited possibilities for a “homelike environment” due to the risk of falls (e.g., carpets and moveable furniture). Small-scale group living concepts should be preferred for people with dementia because these promote greater patient involvement in activities [35].

Higher levels of BPSDs, poor physical function, and cognitive impairment translate into a substantial economic burden [17]. Prior literature has identified higher age [13, 36], poor health [13], and cognitive impairment [13, 37] as factors affecting greater family involvement. We found that agitation cluster symptoms were positively associated with formal direct care time and informal leisure time. A study from the US demonstrated that agitation decreased significantly during the family visits but returned to baseline levels after 30 minutes. The authors proposed that after the caregivers leave, patients cannot fill the void due to cognitive impairment, and thus confusion and agitated behavior occur [38]. Studies also show higher levels of agitation and wandering in physical in-activity [39, 40]. Alternatively, patients with dementia can be sensitive to their surroundings, e.g., intensive conversation during the family visit, long walks, and crowded cafeterias, and thus a moderate level of stimulation is best. The association between lower formal leisure time and poor ADL function raises the question whether some patients are neglected by the NH staff. This may lead to psychosocial and physical unmet needs in this patient group which is also a serious clinical and ethical violation. Research shows that the level of unmet needs is related to both patient characteristics (e.g., higher level of disability and cognitive impairment) and facility factors (e.g., higher patients-to-staff ratio, high turnover etc.) [41]. Non-pharmacological multicomponent interventions are recommended as a first line treatment although, this approach is more resource demanding in people with dementia [42, 43]. Meanwhile, in our and other studies, psychotropic drugs are often prescribed to treat BPSD despite considerable adverse effects [44, 45].

NH patients who need extensive help during ADL tasks are not prioritized for activities because of their complex medical conditions and fear of falling [46, 47]. This supports our findings that reduced physical capacity is related to lower leisure time from the staff. However, higher informal care was associated with lower physical function. These results may imply that caregivers seek for a more tailored and meaningful activity plan. It is essential to include and support family caregivers in developing a personal activity plan and in identifying potential risks of burden related to the patient’s functional decline or challenging behavior. The trend toward greater informal care utilization observed among married patients is mediated by a combination of factors, for instance, a desire to maintain a meaningful relationship or advocacy to ensure good quality of care.

Studies highlight families remaining involved in BPSD management [48] and leisure activities in care homes [49]. Further, enhanced knowledge about caregivers’ involvement in NHs shows that it requires careful planning, training, and commitment from staff and families and a mutual understanding of interest [50]. A good balance between the helper and the professional staff regarding individualized care planning, education, information, and a “care for the carer” strategy may benefit family members’ unfilled needs and improve overall resource utilization in NHs.

### Strengths and limitations

This study used data from a comprehensive sample of NH patients with and without dementia, yielding high generalizability for our findings. The validated primary and secondary outcomes are commonly used in clinical practice and research, allowing comparison with other trials in diverse populations both nationally and globally.

A limitation of this study are the cross-sectional data, hindering us from exploring causal effects. Leisure time should be interpreted with caution due to missing data and taken into consideration when assessing the generalizability of our study results. Estimating direct supervision time even though people with dementia are gathered in common residential areas is one of the RUD-FOCA’s weaknesses, and such time should be estimated using more detailed operationalizations [20]. Another bottleneck is the registration of formal care for bedridden patients who need two or more staff members. Here, care time could have been underestimated and the association between lower physical functioning and higher formal care use might be even stronger. Data were assessed by proxies for both direct one-to-one care and leisure time, and thus were prone to recall bias. Leisure and supervision time might be provided jointly because activities are arranged for many units simultaneously, thus care time in these cases might be overestimated. Further, our study did not include measurements of family members’ demographics or their service satisfaction and expectations, which might explain informal care use in a larger context.

## Conclusions

In this study, we explored which clinical and organizational factors are associated with resource utilization in NHs. We found that patients with higher agitation and psychotropic use, and lower cognitive function receive more care time by NH staff. However, leisure time activities by NH staff are less prioritized in people with lower ADL function. We encourage stakeholders and healthcare professionals to consider these clinical and organizational factors to support NH patients with special demands and value increased family-involvement.

## Supplementary Information

**Additional file 1.**

## Data Availability

The data of the current study are not publicly available due to protection of individual privacy but are available from the corresponding author on reasonable request.

## Abbreviations

ADL: Basic Activities of Daily Living
BPSD: Behavioral and psychological symptoms of dementia
CI: Confidence Interval
COSMOS: COmmunication, Systematic pain assessment and management, Medication review, Organization of activities, and Safety
GLM: Generalized Linear Regression
IADL: Instrumental Activities of Daily Living
MMSE: The Mini-Mental Status Examination
NH: Nursing home
NPI-NH: Neuropsychiatric Inventory – Nursing Home Version
OR: Odds Ratio
PSMS: Physical Self-Maintenance Scale
PwD: People with dementia
RUD - FOCA: Resource Utilization in Dementia – Formal Care tool

## References

[1] El-Hayek YH, Wiley RE, Khoury CP, Daya RP, Ballard C, Evans AR (2019). Tip of the iceberg: assessing the global socioeconomic costs of Alzheimer's disease and related dementias and strategic implications for stakeholders. J Alzheimers Dis.

[2] Gitlin LN, Kales HC, Lyketsos CG (2012). Nonpharmacologic management of behavioral symptoms in dementia. JAMA..

[3] Houttekier D, Vandervoort A, Van den Block L, van der Steen JT, Vander Stichele R, Deliens L (2014). Hospitalizations of nursing home residents with dementia in the last month of life: results from a nationwide survey. Palliat Med.

[4] Afram B, Stephan A, Verbeek H, Bleijlevens MH, Suhonen R, Sutcliffe C (2014). Reasons for institutionalization of people with dementia: informal caregiver reports from 8 European countries. J Am Med Dir Assoc.

[5] Vossius C, Selbæk G, Ydstebø A, Benth J, Godager G, Lurås H (2015). Ressursbruk Og Sykdomsforløp Ved Demens (REDIC) [resource use and disease course in dementia (REDIC)].

[6] Ydstebo AE, Benth JS, Bergh S, Selbaek G, Vossius C (2020). Informal and formal care among persons with dementia immediately before nursing home admission. BMC Geriatr.

[7] Metzelthin SF, Verbakel E, Veenstra MY, Van Exel J, Ambergen AW, Kempen GI (2017). Positive and negative outcomes of informal caregiving at home and in institutionalised long-term care: a cross-sectional study. BMC Geriatr.

[8] Vossius C, Selbæk G, Šaltytė Benth J, Wimo A, Bergh SJIjogp. The use of direct care in nursing home residents: a longitudinal cohort study over 3 years. Int J Geriatric Psychiatry. 2018; 34(2):337–51. 10.1002/gps.5026 PMC6590302 30430646

[9] Røen I, Selbæk G, Kirkevold Ø, Engedal K, Testad I, Bergh S (2017). Resourse use and disease Couse in dementia - nursing home (REDIC-NH), a longitudinal cohort study; design and patient characteristics at admission to Norwegian nursing homes. BMC Health Serv Res.

[10] Hayward JK, Gould C, Palluotto E, Kitson EC, Spector A. Family involvement with care homes following placement of a relative living with dementia: a review. Ageing Soc. 2021:1–46. 10.1177/14713012211046595 PMC8811321 34894796

[11] Paulus AT, Raak A, Keijzer F (2005). Informal and formal caregivers' involvement in nursing home care activities: impact of integrated care. J Adv Nurs.

[12] Puurveen G, Baumbusch J, Gandhi P (2018). From family involvement to family inclusion in nursing home settings: a critical interpretive synthesis. J Fam Nurs.

[13] Roberts AR, Ishler KJ, Adams KB (2020). The predictors of and motivations for increased family involvement in nursing homes. Gerontologist..

[14] Powell C, Blighe A, Froggatt K, McCormack B, Woodward-Carlton B, Young J (2018). Family involvement in timely detection of changes in health of nursing homes residents: a qualitative exploratory study. J Clin Nurs.

[15] Nordberg G, Wimo A, Jönsson L, Kåreholt I, Sjölund BM, Lagergren M (2007). Time use and costs of institutionalised elderly persons with or without dementia: results from the Nordanstig cohort in the Kungsholmen project—a population based study in Sweden. Int J Geriatr Psychiatry.

[16] Buylova Gola A, Morris S, Candy B, Davis S, King M, Kupeli N (2020). Healthcare utilization and monetary costs associated with agitation in UK care home residents with advanced dementia: a prospective cohort study. Int Psychogeriatr.

[17] Angeles RC, Berge LI, Gedde MH, Kjerstad E, Vislapuu M, Puaschitz NG (2021). Which factors increase informal care hours and societal costs among caregivers of people with dementia? A systematic review of resource utilization in dementia (RUD). Heal Econ Rev.

[18] Husebo BS, Ballard C, Aarsland D, Selbaek G, Slettebo DD, Gulla C (2019). The effect of a multicomponent intervention on quality of life in residents of nursing homes: a randomized controlled trial (COSMOS). J Am Med Dir Assoc.

[19] Husebo BS, Flo E, Aarsland D, Selbaek G, Testad I, Gulla C, et al. COSMOS-improving the quality of life in nursing home patients: protocol for an effectiveness-implementation cluster randomized clinical hybrid trial. Implement Sci. 2015;10(1):1-13. 10.1186/s13012-015-0310-5 PMC4572450 26374231

[20] Luttenberger K, Graessel E (2010). Recording care time in nursing homes: development and validation of the "RUD-FOCA" (resource utilization in dementia-formal care). Int Psychogeriatr.

[21] Wimo A, Gustavsson A, Jonsson L, Winblad B, Hsu MA, Gannon B (2013). Application of resource utilization in dementia (RUD) instrument in a global setting. Alzheimers Dement.

[22] Perneczky R, Wagenpfeil S, Komossa K, Grimmer T, Diehl J, Kurz A (2006). Mapping scores onto stages: mini-mental state examination and clinical dementia rating. Am J Geriatr Psychiatry.

[23] Selbaek G, Kirkevold O, Sommer OH, Engedal K (2008). The reliability and validity of the Norwegian version of the neuropsychiatric inventory, nursing home version (NPI-NH). Int Psychogeriatr.

[24] Lawton MP, Brody EM (1969). Assessment of older people: self-maintaining and instrumental activities of daily living. The Gerontologist.

[25] ATC/DDD index. Oslo: WHO collaborating Centre for Drug Statistics Methodology; 2015.

[26] Dunn G, Mirandola M, Amaddeo F, Tansella M (2003). Describing, explaining or predicting mental health care costs: a guide to regression models. Methodological review. Br J Psychiatry.

[27] Neelon B, O'Malley AJ, Smith VA (2016). Modeling zero-modified count and semicontinuous data in health services research part 1: background and overview. Stat Med.

[28] Akaike H (1974). A new look at the statistical model identification. IEEE Trans Autom Control.

[29] Døhl Ø, Garåsen H, Kalseth J, Magnussen J (2014). Variations in levels of care between nursing home patients in a public health care system. BMC Health Serv Res.

[30] Kjøs BØ, Havig AK (2016). An examination of quality of care in N orwegian nursing homes–a change to more activities?. Scand J Caring Sci.

[31] Kirkevold O, Engedal K (2006). The quality of care in Norwegian nursing homes. Scand J Caring Sci.

[32] Drennan VM, Ross F (2019). Global nurse shortages-the facts, the impact and action for change. Br Med Bull.

[33] Ribeiro O, Araújo L, Figueiredo D, Paúl C, Teixeira L (2022). The Caregiver Support Ratio in Europe: Estimating the Future of Potentially (Un) Available Caregivers.

[34] Førsund LH, Ytrehus S (2018). Finding a place to connect: a qualitative study exploring the influences of the physical and social environments on spouses’ opportunities to maintain relationships when visiting a partner with dementia living in long-term care. Dementia..

[35] Anderiesen H, Scherder EJ, Goossens RH, Sonneveld MH (2014). A systematic review–physical activity in dementia: the influence of the nursing home environment. Appl Ergon.

[36] Gaugler JE, Anderson K, Zarit S, Pearlin L (2004). Family involvement in nursing homes: effects on stress and well-being. Aging Ment Health.

[37] Cohen LW, Zimmerman S, Reed D, Sloane PD, Beeber AS, Washington T (2014). Dementia in relation to family caregiver involvement and burden in long-term care. J Appl Gerontol.

[38] Martin-Cook K, Hynan L, Chafetz PK, Weiner MF (2001). Impact of family visits on agitation in residents with dementia. Am J Alzheimers Dis Other Dement.

[39] Scherder EJA, Bogen T, Eggermont LHP, Hamers JPH, Swaab DF (2010). The more physical inactivity, the more agitation in dementia. Int Psychogeriatr.

[40] Traynor V, Veerhuis N, Johnson K, Hazelton J, Gopalan S (2018). Evaluating the effects of a physical activity on agitation and wandering (PAAW) experienced by individuals living with a dementia in care homes. J Res Nurs.

[41] National Academies of Sciences Engineering, and Medicine (2022). The National Imperative to Improve Nursing Home Quality: Honoring Our Commitment to Residents, Families, and Staff.

[42] Livingston G, Huntley J, Sommerlad A, Ames D, Ballard C, Banerjee S (2020). Dementia prevention, intervention, and care: 2020 report of the lancet commission. Lancet..

[43] Ballard C, Corbett A, Orrell M, Williams G, Moniz-Cook E, Romeo R (2018). Impact of person-centred care training and person-centred activities on quality of life, agitation, and antipsychotic use in people with dementia living in nursing homes: a cluster-randomised controlled trial. PLoS Med.

[44] Gedde MH, Husebo BS, Mannseth J, Kjome RL, Naik M, Berge LI (2021). Less is more: the impact of deprescribing psychotropic drugs on behavioral and psychological symptoms and daily functioning in nursing home patients. Results from the cluster-randomized controlled COSMOS trial. Am J Geriatr Psychiatry.

[45] Birkenhager-Gillesse EG, Kollen BJ, Achterberg WP, Boersma F, Jongman L, Zuidema SU (2018). Effects of psychosocial interventions for behavioral and psychological symptoms in dementia on the prescription of psychotropic drugs: a systematic review and Meta-analyses. J Am Med Dir Assoc.

[46] Guralnik JM, LaCroix AZ, Abbott RD, Berkman LF, Satterfield S, Evans DA (1993). Maintaining mobility in late life. I. Demographic characteristics and chronic conditions. Am J Epidemiol.

[47] Schulz C, Lindlbauer I, Rapp K, Becker C, Konig HH (2017). Long-term effectiveness of a multifactorial fall and fracture prevention program in Bavarian nursing homes: an analysis based on health insurance claims data. J Am Med Dir Assoc.

[48] Tjia J, Lemay CA, Bonner A, Compher C, Paice K, Field T (2017). Informed family member involvement to improve the quality of dementia care in nursing homes. J Am Geriatr Soc.

[49] Smit D, De Lange J, Willemse B, Pot AM (2017). Predictors of activity involvement in dementia care homes: a cross-sectional study. BMC Geriatr.

[50] Tasseron-Dries PEM, Smaling HJA, Doncker S, Achterberg WP, van der Steen JT (2021). Family involvement in the Namaste care family program for dementia: a qualitative study on experiences of family, nursing home staff, and volunteers. Int J Nurs Stud.

[51] World Medical Association (2001). World medical association declaration of Helsinki. Ethical principles for medical research involving human subjects. Bull World Health Organ.

