# Supplementary material for: Factors associated with formal and informal resource utilization in nursing home patients with and without dementia: cross-sectional analyses from the COSMOS trial
Source: BMC Health Serv Res. 2022 Nov 2;22:1306. doi: 10.1186/s12913-022-08675-y (PMC9628082; doi:10.1186/s12913-022-08675-y)
Supplement: Supplementary file 1 — Additional file 1. [file 12913_2022_8675_MOESM1_ESM.docx]

**Appendix A** Comparison of clinical factors between patients with complete and missing data on total leisure time

|  | **Complete data**  **(N=226)** | **Missing data**  **(N=296)** | **p-value** |
| --- | --- | --- | --- |
| Age in years, mean (SD) | 86.1 (7.8) | 87.1 (7.2) | .14 |
| Female gender, n (%) | 165 (73.0) | 223 (75.3) | .54 |
| Married, n (%) | 46 (21.1) | 72 (27.4) | .11 |
| Psychotropic medication^a^ regular use, 1 or more drug, n (%) | 163 (72.1) | 219 (74.0) | .63 |
| Polypharmacy^b^, n (%) | 190 (84.1) | 221 (74.7) | **<.05** |
| MMSE^c^, mean (SD) | 11.6 (7.8) | 10.5 (7.7) | .16 |
| PSMS^d^, mean (SD) | 16.7 (5.5) | 17.8 (5.3) | **<.05** |
| **NPI clusters**^e^**, mean (SD)** |  |  |  |
| Mood cluster (0-60) | 8.7 (9.9) | 7.3 (9.5) | **<.05** |
| Psychosis cluster (0-24) | 2.7 (5.0) | 2.4 (4.7) | .65 |
| Agitation cluster (0-48) | 7.7 (10.5) | 6.7 (9.2) | .31 |
| Table legend: N=total sample; n=number of patients; SD=standard deviation. Tested with independent samples t-tests for normally distributed continuous variables, Mann–Whitney U-test for nonnormally distributed continuous variables, and Pearson chi-squared tests for categorical variables.  ^a^According to The Anatomical Therapeutic Chemical Index (antipsychotics, anxiolytics, hypnotics/sedatives, antidepressants, and anti-dementia agents).  ^b^5 or more drugs regularly.  ^c^MMSE: Mini-Mental State Examination [range 0-30], a higher score indicates better cognition.  ^d^PSMS – Physical Self-Maintenance Scale [range 0-30], a higher score indicates lower functional capacity.  ^e^NPI-NH: Neuropsychiatric Inventory – Nursing Home Version. Mood cluster (depression, anxiety, apathy, sleep, and appetite); agitation cluster (agitation, disinhibitions, irritability, and aberrant motor behavior), and psychosis cluster (hallucinations and delusions). | | | |
